# Deep Sparse Learning for Automatic Modulation Classification Using Recurrent Neural Networks

**DOI:** 10.3390/s21196410

**Published:** 2021-09-25

**Authors:** Ke Zang, Wenqi Wu, Wei Luo

**Affiliations:** 1 College of Biomedical Engineering and Instrument Science, Yuquan Campus, Zhejiang University, 38 Zheda Road, Hangzhou 310027, China; martinzang@zju.edu.cn (K.Z.); winkywow@zju.edu.cn (W.W.); 2 Department of Biomedical Engineering, The Chinese University of Hong Kong, Shatin, New Territories, Hong Kong, China

**Keywords:** automatic modulation classification, deep sparse learning, recurrent neural networks

## Abstract

Deep learning models, especially recurrent neural networks (RNNs), have been successfully applied to automatic modulation classification (AMC) problems recently. However, deep neural networks are usually overparameterized, i.e., most of the connections between neurons are redundant. The large model size hinders the deployment of deep neural networks in applications such as Internet-of-Things (IoT) networks. Therefore, reducing parameters without compromising the network performance via sparse learning is often desirable since it can alleviates the computational and storage burdens of deep learning models. In this paper, we propose a sparse learning algorithm that can directly train a sparsely connected neural network based on the statistics of weight magnitude and gradient momentum. We first used the MNIST and CIFAR10 datasets to demonstrate the effectiveness of this method. Subsequently, we applied it to RNNs with different pruning strategies on recurrent and non-recurrent connections for AMC problems. Experimental results demonstrated that the proposed method can effectively reduce the parameters of the neural networks while maintaining model performance. Moreover, we show that appropriate sparsity can further improve network generalization ability.

## 1. Introduction

Automatic modulation classification (AMC) refers to the automatic recognition of the modulation category of the received signal. This technology is widely used in spectrum management and interference recognition, etc. [1,2]. With the rapid development of wireless communication in recent years, the modulation types of signals has become more complex and diverse, which makes AMC a crucial technique in crowded radio environments.

Initially, the likelihood-based method was used to solve the modulation classification problem [3,4]. Although the methods based on likelihood can reduce the probability of mismatch, they usually suffer from high computational complexity, and their self-based theoretical system models are difficult to match with practical scenarios. To alleviate the computation overhead in practice, feature-based method [5,6,7] for AMC arose in response to the proper time and conditions. Traditional feature-based methods mainly consist of manually extracted features and appropriate classifiers. The feature-based approach became the mainstream because it is robust relative to different signals and has better generalization ability. With the development of Big Data and the improvement of computing power, deep neural networks has shown its powerful potential in many fields, such as computer vision [8] and natural language processing [9]. Among these models, recurrent neural networks (RNNs) [10] have been playing an essential role in tasks involving sequential data due to their ability to find the dependencies between data located in different parts of a sequence. For AMC problems, the manually designed features in traditional methods are usually extracted both locally and globally from the observed signal, which is exactly what RNNs are good at. Moreover, it is often observed that manually designing features may result in loss of information that is essential for accurate classification [11]. Therefore, researchers have attempted to use RNNs such as long short-term memory (LSTM) [12] for higher accuracy in AMC problems [13,14,15]. However, being overparameterized is a widely recognized property of deep neural networks [16,17]. It is difficult to apply deep neural networks into the edge devices [18,19], such as Internet-of-Thing(IoT) devices, which are usually equipped with limited device memories [20]. Therefore, removing the redundant connections of deep learning models while maintaining their performance is often desirable since it can alleviate computational and storage burdens.

Sparse learning is an efficient technique for training a sparsely connected neural network. Neural network pruning, the task of reducing the size of a network by removing parameters, has been the subject of numerous studies in recent years. Pruning a neural network amounts to removing its superfluous connections while maintaining model performance as much as possible [18,21]. The sparsity of a deep learning network is determined by the proportion of zeros in its trainable parameters θ. A sparse structure is usually obtained by multiplying the θ element wisely by a binary mask ***m*** of the same size.

(1) $$\theta^{sparse}=\theta ⊙m.$$

**Figure 1:**
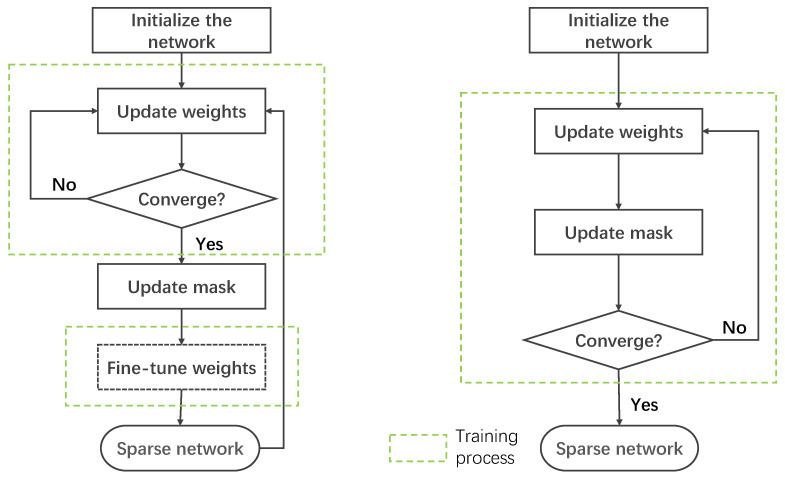
Iterative pruning (**left**) and one-shot pruning (**right**).

Here, ⊙ stands for element-wise multiplication. For many years, people have generally believed that training a dense, overparameterized network in advance is the key to effective subsequent sparseness [22]. Accordingly, certain methods are needed to remove redundant parameters without significantly affecting the model performance. Most of the currently used algorithms for generating sparse masks begin with a dense model, and then they increase the sparsity of the model through the network pruning. Pruning methods can be divided into two categories: iterative pruning [23,24,25,26] and one-shot pruning [22,27,28]. As shown in Figure 1, an iterative pruning method first trains a model until it converges. Then, the sparse mask m will be updated based on specific criteria that vary across different methods. This process is repeated until the model sparsity meets the requirements. Han et al. introduced a method to reduce the storage and computation of neural networks by an order of magnitude via removing the unimportant connections [23]. Frankle et al. proposed the Lottery Ticket Hypothesis, that is, a dense, randomly initialized feedforward network with separate training containing subnetworks (winning tickets) that can achieve similar test accuracy as the original network under a similar number of iterations [26]. In addition, they presented an iterative pruning algorithm to identify winning tickets on the MNIST and CIFAR10 datasets. On the other hand, the model training process in one-shot pruning algorithms is interleaved with pruning steps. Thus, a sparsely connected model can be obtained within one training process. In [27], the authors prune the network through the $L_{0}$ norm regularization that allows for straightforward and efficient learning of model structures with stochastic gradient descent. The single-shot network pruning method [22] prunes a given network once at initialization prior to training. After pruning, a network is trained in the standard manner.

In existing methods, pruning is usually performed by using an iterative finetuning process, or with a pruning scheme designed heuristically, or with the addition of hyperparameters, thereby undermining their utility. In this work, we present a new approach that prunes a given network based on the statistics of the weight magnitude and gradient momentum without iterative training. Unlike other pruning algorithms that were applied only to feedforward neural networks (FNNs), such as multilayer perceptrons (MLPs) and convolutional neural networks (CNNs) on non-sequential data, the proposed method in this paper is validated on both feedforward and recurrent neural architectures. The main contributions of this paper are as follows:

(1) A novel one-shot neural network pruning algorithm based on weight magnitude and gradient momentum is proposed to produce sparse RNNs for solving AMC problems without compromising model performance. Specifically, we demonstrate that it is crucial to retain non-recurrent connections while pruning RNNs.
(2) In addition to the sequential AMC problem, the efficiency of the proposed method is also validated on non-sequential dataset, including MNIST and CIFAR10, with feedforward neural networks.
(3) The experimental results reveal that the proposed pruning method can serve as a regularization technique as the resulting sparse models can outperform their dense counterparts even with high-level sparsity.

## 2. Methods

### 2.1. Notation

Bold numbers such as 1 and 0 denote vectors consist of the corresponding numbers. The element-wise multiplication is denoted by ⊙, and the convolution operation is denoted by ∗. The sigmoid activation function σ used extensively in deep learning models is defined as $\sigma \left(x\right)=\frac{1}{1+e^{-x}}$, and the hyperbolic function tanh is defined as tanh(x)=2σ(2x)−1.

### 2.2. Recurrent Neural Networks

Recurrent neural networks [29,30,31], particularly with gated cells such as LSTMs [12] and gated recurrent units (GRUs) [32], are perhaps the most popular architectures for modeling temporal sequences. The LSTM reads from and writes to its internal states by using a gating mechanism, which allows information to pass selectively. There are three different kinds of gating units inside an LSTM cell, namely the write, read, and forget gates. The write and read gates are used to filter out useless information flowing in and out of the recurrent cells, respectively, while the forget gates can selectively erase old memories.

**Figure 2:**
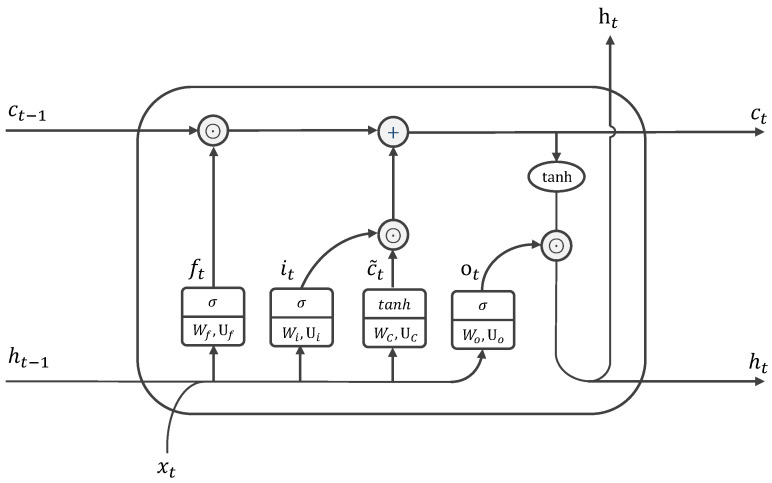
Long short-term memory [14].

These three gates are realized by the gating mechanism, as shown in Figure 2, and are formulated as follows.

(2a) $$\begin{array}{cc} i_{t} & =\sigma (W_{i}h_{t-1}+U_{i}x_{t}+b_{i}), \end{array}$$

(2b) $$\begin{array}{cc} o_{t} & =\sigma (W_{o}h_{t-1}+U_{o}x_{t}+b_{o}), \end{array}$$

(2c) $$\begin{array}{cc} f_{t} & =\sigma (W_{f}h_{t-1}+U_{f}x_{t}+b_{f}). \end{array}$$

The candidate memory ${\tilde{c}}_{t}$ to be written is defined as the following.

(3) $${\tilde{c}}_{t}=\tanh\left(W_{c}h_{t-1}+U_{c}x_{t}+b_{c}\right).$$

The new memory $c_{t}$ and the information flowing out the cell $h_{t}$ are then given by the following.

(4) $$c_{t}=c_{t-1}⊙f_{t}+{\tilde{c}}_{t}⊙i_{t},$$

(5) $$h_{t}=o_{t}⊙\tanh\left(c_{t}\right).$$

GRU is another popular recurrent architecture based on gate units, which was first introduced by Chung et al. in 2014 [32]. It can be seen as a variation of LSTM that explicitly couples write and forget gates.

**Figure 3:**
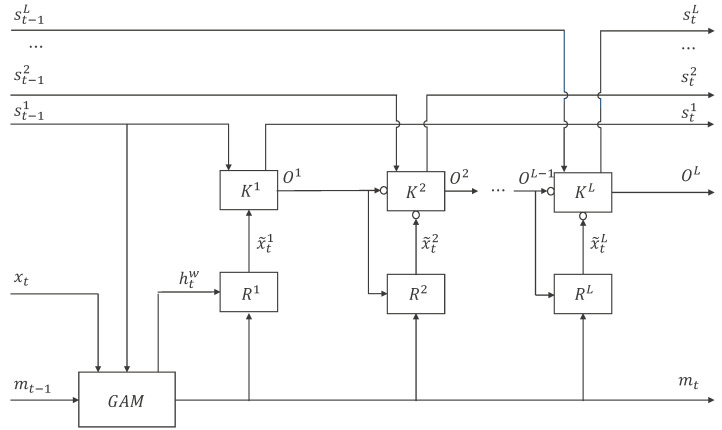
Diagram of the hierarchical recurrent neural network with grouped auxiliary memory architecture. Inputs conveyed by arrows with ∘ will be concatenated together [14].

Another RNN we used in this paper is a hierarchical RNN with grouped auxiliary memory named GAM-HRNN [14]. As shown in Figure 3, the main framework of the network is a hierarchical structure with other RNNs as the kernel. At each time step, due to the group distributed mechanism, the corresponding part of the unit of state is overwritten, while the other parts change slightly or remain unchanged. In this manner, useful information can be saved without overwriting the long-term memory. After the auxiliary memory has been updated, the state of each layer in the hierarchical structure is updated sequentially by using the information selectively read from the auxiliary memory and the state passed from the previous time step. In this manner, the network provides a shortcut in time and space, which is good for confronting conflicts between short and long periods of information and preserving long-term information. Formulation details can be found in [14].

Note that the learnable weights in recurrent units are denoted by W and U, representing the recurrent and non-recurrent connections, respectively. We used different pruning strategies for these two kinds of weights in the experiments of this paper.

### 2.3. Pruning Method

Given a dataset $D^{train}={\left(x_{i},y_{i}\right)}_{i=1}^{N}$, the objective function can be formulated as follows.

(6) $$E\left(F\left(\cdot ,\theta \right);D^{train}\right)=\frac{1}{N}\sum_{i=1}^{N}L\left(y_{i},F\left(x_{i},\theta \right)\right)).$$

Here, L is the loss function such as cross-entropy, and θ denotes the parameters of network F(·,θ).

The main hypothesis behind the neural network sparsity literature is that neural networks are usually overparameterized, implying that most elements in the weight vector $\theta^{*}$ of a converged model $F(\cdot ,\theta^{*})$ are redundant. Thus, comparable performance can be achieved by using a smaller network [33], e.g., the sparse version of this model $F(\cdot ,m⊙\theta^{*})$. Here, ***m*** is a sparse mask. To this end, the objective is to learn a sparse network while maintaining the accuracy of its dense counterpart as much as possible. The sparse mask can be generated via network pruning. A part of the pruning algorithms quantifies the importance of network connections based on their magnitude [23,26]. However, these methods will also remove connections that can greatly reduce the loss after being updated, yet they are not significant in magnitude. This can be circumvented by considering the weight gradient $G_{t}=\nabla_{\theta }E$ as another factor to measure the connection sensitivity [22,28,34]. In practice, deep learning models are always trained using the stochastic gradient descend algorithm; thus, the gradient momentum is always used to estimate the global gradient calculated on the entire dataset.

(7) $$v_{t}=\gamma v_{t-1}+\left(1-\gamma \right)\nabla_{\theta }E\left(\theta \right).$$

Here, γ is usually set to a value that is less than but close to 1, e.g., 0.9.

In this paper, we propose a novel one-shot neural network pruning algorithm based on both magnitude and gradient momentum of learnable parameters. The algorithm is described in Algorithm 1. Previous investigation has already shown that removing the connections with small magnitude can yield sparse network without sacrificing performance. Thus, in the proposed method, we directly preserve the weights of large magnitudes (Algorithm 1 line 11). In order to preserve the weak connections that may potentially contribute to reducing training loss, we also used the gradient momentum to estimate the importance of each weight. Assume that N connections should be pruned in each iteration, we first select δ· N connections with the smallest magnitude as candidates. Here, δ(>1) is a hyper-parameter. We then select N connections with the lowest score of importance and remove them from the network by updating the sparse mask. The score of importance for each parameter $\theta_{i}$ is defined as follows:

(8) $$s_{i}=\alpha \cdot |\theta_{i}\left|+\left(1-\alpha \right)\cdot \right|v_{i}|,$$

where $v_{i}$ is the gradient momentum of the *i*-th parameter $\theta_{i}$, and  α is another hyper-parameter.
**Algorithm 1** The proposed method**Require:**
Training set $D^{train}=\left(x_{i},y_{i}\right)$
**Require:**
Network F with parameters θ
**Require:**
Pruning interval K, hyper-parameter for calculating momentum γ, hyper-parameter for pruning δ and α, pruning rate *p*.
1:Initialize the parameters $\theta \leftarrow \theta_{0}$;2:Initialize the momentum v←0;3:Initialize the parameter mask: **m**←1;4:**repeat**5:    **for** n=1 to K **do**6:        Generate data batch: $D^{batch}∼D^{train}$;7:        Update θ:$\theta \leftarrow update(\theta ⊙m,D^{batch})$;8:        Update momentum using Equation (7);9:    **end for**10:    Get number of connections to be pruned N=p·sum(m);11:    Get the candidate mask based on magnitude: $m_{c}\leftarrow$ getCandidateMask(δ·N,θ);12:    Calculate score of importance *s* using Equation (8);13:    Select connections to be pruned among candidates based on score $m_{s}\leftarrow$ get Connections To Prune($s,m_{c}$);14:    Update sparse mask m← update($m_{s}$);15:**until** meeting training termination condition

Note that in the early stage of the proposed algorithm, the network can be trained without pruning for several iterations for warming up, which may improve the final performance in some cases. In this paper, the training process is always terminated when a certain degree of sparsity has been met.

## 3. Experimental Results and Discussions

In this section, we evaluate the performance of the proposed method on the standard MNIST and CIFAR10 datasets that are always used as benchmark datasets for pruning tasks by comparing with other pruning methods. Then, we used the proposed method for AMC problems on the standard RadioML 2016.10a dataset via RNNs comparing with other classification methods.

### 3.1. Experimental Configuration

We used Xavier uniform initalizer [35] for all weights and Adam optimizer [36] for the training processes. The models were implemented using Tensorflow [37]. All experiments were repeated 10 times.

#### 3.1.1. MNIST and CIFAR10 Datasets

The proposed method in this paper is first compared with other sparse learning techniques including a magnitude-based pruning algorithm proposed in [23] and a rewinding-after-pruning training scheme used to find the ‘lottery ticket’ presented in [26]. All pruning methods to be compared are performed on the Lenet-300-100 [38] model for the MNIST dataset and a two-layer CNN for the CIFAR10 dataset. The Lenet-300-100 model is a fully connected network with two hidden layers consisting of 300 and 100 neurons, respectively. The two-layer CNN includes two convolutional layers and a pooling layer followed by two fully connected layers and an output layer. We denote this model as Conv2 in this paper. Other details of these two feedforward neural networks can be found in [26]. We adopted the original setups described in the corresponding paper to configure the pruning algorithms to be compared. The hyperparameters used in the proposed method are listed in Table 1.

#### 3.1.2. RadioML 2016.10a Dataset

For AMC problems, we verified the effectiveness of our method by mainly using an open modulated classification dataset named RadioML2016.10a [39]. There are 220,000 RF signals modulated by three analog and eight digital modulation types. Specifically, analog modulation methods include wide band FM (AM-FM), single-sideband AM (AM-SSB), and wideband FM (WB-FM) and digital modulation methods include quadrature phase-shift keying (QPSK), eight phase-shift keying (8PSK), quadrature amplitude modulation 16 (QAM16), quadrature amplitude modulation 64 (QAM64), cover binary phase-shift keying (BPSK), continuous phase frequency-shift keying (CPFSK), Gauss frequency-shift keying (GFSK), and pulse-amplitude modulation four (PAM4). Each signal is 128 in length and samples per symbol is eight. The signal-to-noise ratio (SNR) is evenly distributed from −20 dB to 18 dB at intervals of 2 dB. Radio channel including time varying multi-path fading, random walk drifting, and non-ideal effects covering carrier frequency offset oscillator drift, etc., are well-characterized. More details can be found in [39].

The proposed method was used to prune recurrent neural architectures including LSTM, GRU, and GAM-HRNN. Three pruning methods were also tested on GAM-HRNN model. All of these recurrent models have two hidden layers. The normalized amplitude and phase of the signal are obtained from the original IQ data. We set the forget gate bias to 1.0 for LSTM. Details regarding the model hyperparameters can be found in [14]. Performances of densely connected neural networks including sequential convolutional recurrent neural network (SCRNN) and GAM-HRNN are also reported. All models have roughly the same number of parameters. For RNNs, we only prune the recurrent connections mentioned in 2.1. Details regarding the prune hyperparameters are listed in Table 1.

### 3.2. Results on Standard MNIST and CIFAR10 Datasets

**Figure 4:**
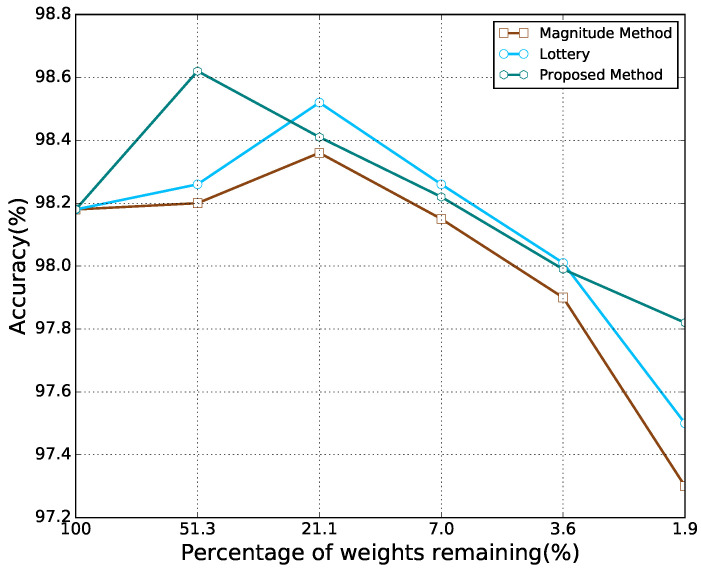
Classification accuracy for different percentages of weights remaining from Lenet-300-100 on MNIST.

**Figure 5:**
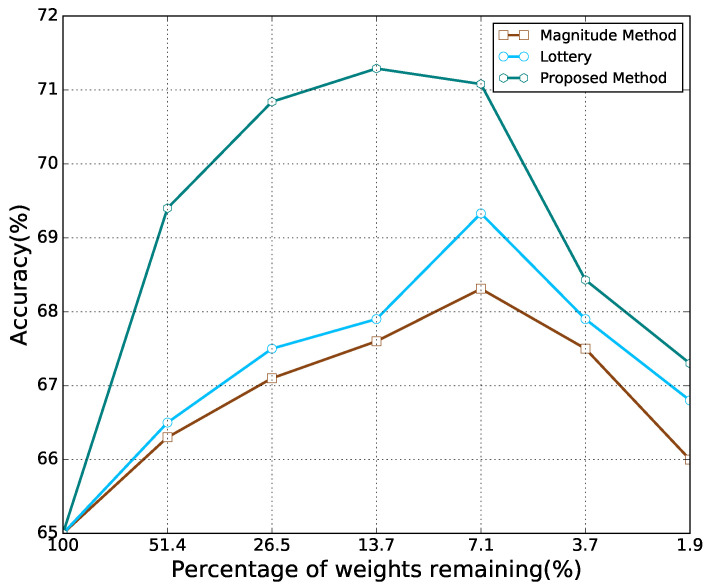
Classification accuracy for different percentages of weights remaining from Conv2 on CIFAR10.

For the MNIST dataset, it can be observed from Figure 4 that for all the methods, as the percentage of weights remaining decreased, the accuracy first increased and then decreased. At a high percentage of weights remaining, the proposed method performed better than other methods. However, as the percentage of weights remaining decreased, the accuracy of the proposed method was observed to be lower than that of the lottery hypothesis. When the percentage of remaining weights further decreased, the proposed method was found to exhibit better performance than others again. As shown in Figure 5, the results of CIFAR10 are similar to those of MNIST. However, on the CIFAR10 dataset, the proposed method performed consistently better than other methods considering the percentage of remaining weights.

The highest accuracy of each model achieved by each pruning method is listed in Table 2. The results demonstrate that a neural network with a certain degree of sparsity may outperform its dense counterpart, and the proposed method in this paper always yields a higher performance boost for each model on each task.

### 3.3. Results on RadioML 2016.10a Dataset

In this section, we discuss the performance of the proposed method for recurrent neural architectures for AMC problems.

**Figure 6:**
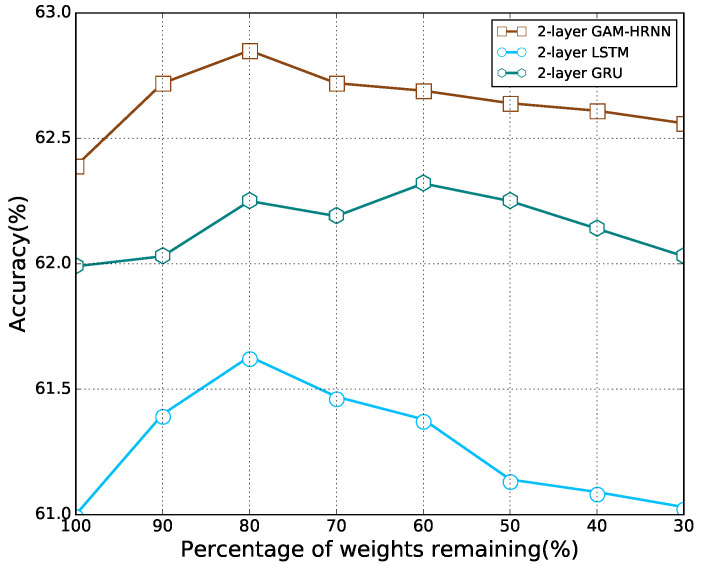
Classification accuracy for the proposed pruning method on three RNN models.

**Figure 7:**
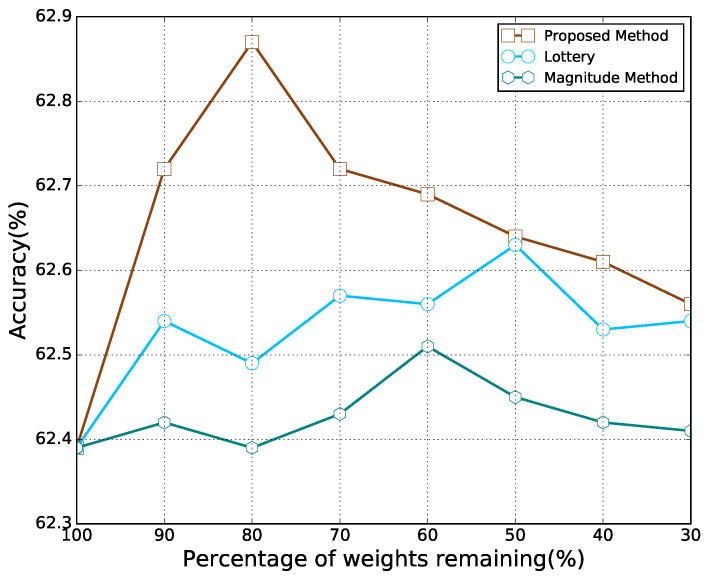
Classification accuracy for different pruning methods on GAM-HRNN model.

**Figure 8:**
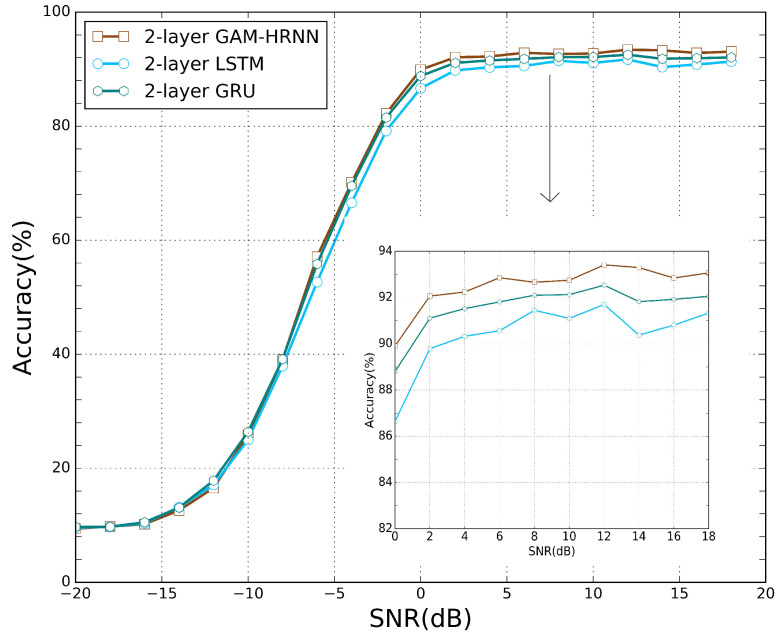
Classification accuracy for the proposed method on three RNN models at different SNRs.

We first apply the proposed pruning method to remove the recurrent connections of RNNs used in [14], including LSTM, GRU, and GAM-HRNN. The classification accuracies with different percentages of remaining weights are presented in Figure 6. It can be observed that as the model parameters decreased, the accuracy of the model first increased and then decreased, which is similar to the feedforward cases. With a certain degree of sparsity, each model can outperform its dense counterpart. Note that the percentage of weights remaining corresponding to the best accuracy for each model is different. Moreover, all recurrent models are still able to beat their dense counterparts with most of their connections being pruned, especially for GAM-HRNN. Classification accuracy for different pruning methods on GAM-HRNN model is presented in Figure 7. We can find that the proposed method also achieve better performance on AMC problems compared to other pruning methods. The performances of other methods on AMC problems are also compared. Table 3 reports the corresponding average accuracy for all SNRs, and the SNR ranges from 0 to 20 dB. The dash symbol in Table 3 indicates that the metric was not reported in the corresponding paper. It can be observed that the sparse GAM-HRNN model produced by the proposed method outperforms the previous state-of-the-art model on both two metrics. Meanwhile, Figure 8 shows the classification accuracy for the proposed method on three RNN models at different SNRs. We can observe that all three models perform poorly at low SNR. However, the advantage of GAM-HRNN over the other two models becomes more obvious as SNR increases.

**Figure 9:**
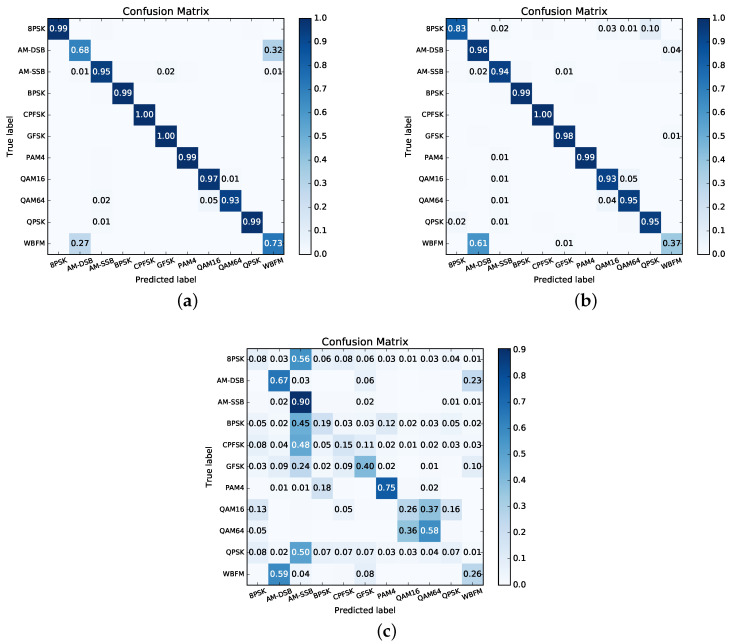
Confusion matrices of 2-layer pruned GAM-HRNN model on RadioML 2016.10a dataset at (**a**) 18 dB SNR, (**b**) 0 dB SNR, and (**c**) −8 dB SNR.

The confusion matrices at three SNRs (18 dB, 0 dB, and −8 dB) for pruned GAM-HRNN with the best accuracy using the proposed method is shown in Figure 9. Even at high SNR, the network cannot distinguish am-DSB and WBFM signals well. This can be attributed to the small observation window (0.64 ms of modulated speech per example) and low information rate with frequent silence between words [43]. Meanwhile, the network also has a certain ambiguity to distinguish QAM16 and QAM64. However, this problem has been alleviated compared to [14] since the proposed method improves the network generalization ability.

**Figure 10:**
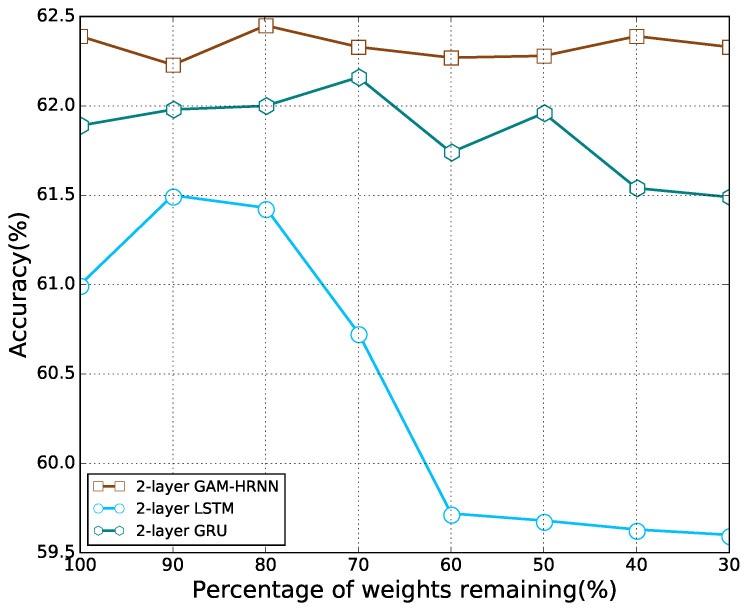
Classification accuracy for the proposed pruning method on three RNN models (both recurrent and non-recurrent connections are pruned).

As mentioned in 2.1, recurrent models have recurrent connections and non-recurrent connections. Figure 6 shows the results of the proposed pruning algorithm by removing only the recurrent connections. The importance of performing this step is illustrated in Figure 10, which shows the consequences of pruning both the recurrent and non-recurrent connections. We can observe that LSTM suffers from an obvious performance decrease as the connections being pruned. As for GRU and GAM-HRNN, the performances are not as good as those shown in Figure 6. Therefore, we conjecture that removing non-recurrent weights that connect input neurons to recurrent units at each time step may result in inefficient feature extraction, resulting in worse model performance. On the other hand, pruning recurrent weights appropriately can facilitate RNN memory transmission.

## 4. Conclusions

In this paper, we present a sparse learning algorithm for RNNs on AMC problems based on the statistics of weight magnitude and gradient momentum. We demonstrate experimentally that non-recurrent connections should be retained during pruning. The proposed method can alleviate the computational and storage burden for recurrent models, facilitating their hardware implementations on devices with limited resources. Furthermore, experimental results also show that the proposed method can produce a neural model with a certain degree of sparsity that outperforms its dense counterpart. The efficiency of the proposed method is verified on both feedforward and recurrent neural architectures.

Our future work includes many aspects. For example, as the surviving neural connections are updated during training, the gradient of training loss with respect to the removed connections may become larger. Such connections can also contribute to reducing the training loss efficiently. Thus, mechanisms to restore the pruned weights can be considered. On the other hand, the proposed method produces unstructured sparsity other than structured sparsity [24]. Hence, the resulting sparse networks are currently not supported in terms of being accelerated by hardware. For this reason, the proposed method can be further extended to produce structured sparsity.

## Figures and Tables

**Table 1:** Pruning hyper-parameters for Lenet-300-100, Conv2, and RNNs.

	Lenet-300-100 (MNIST)	Conv2 (CIFAR10)	RNNs (RadioML)
Batch size	100	64	400
Warm up epochs	1	5	10
Prune frequency	Once per epoch	Once per epoch	Once per epoch
Prune rate p	0.005	0.002	0.002
α	0.2	0.3	0.3
γ	0.4	0.3	0.3
δ	2	2	2

**Table 2:** Best accuracy of each model on MNIST and CIFAR10 (%).

	Lenet-300-100 on MNIST Accuracy (%)	Conv2 on CIFAR10 Accuracy (%)
Unpruned baseline	98.16 ± 0.06	65.09 ± 0.045
Magnitude-based [23]	98.40	68.31
Lottery [26]	98.52	69.33
Proposed method	98.62 ± 0.067	71.29 ± 0.059

**Table 3:** Average accuracy for all SNRs (AccAS) and for SNR ranges from 0 dB to 20 dB (AccASH) (%).

	AccAS		AccASH	
	Original	Pruned	Original	Pruned
2-layer GAM-HRNN	62.47 [14]	62.87 ± 0.077	92.2 [14]	92.45 ± 0.083
2-layer LSTM	60.8 ± 0.073	61.12 ± 0.11	90 [13]	90.81 ± 0.09
2-layer GRU	61.68 ± 0.087	62.06 ± 0.073	91.13 ± 0.069	91.59 ± 0.063
Cross model [40]	62.41	-	-	-
Multipath CNN [41]	-	-	90.7	-
Multitask CNN [42]	59.43	-	-	-
SCRNN [15]	-	-	92.1	-

## Data Availability

Not applicable.
